# Comparative analysis of IOL power calculations in postoperative refractive surgery patients: a theoretical surgical model for FS-LASIK and SMILE procedures

**DOI:** 10.1186/s12886-023-03164-0

**Published:** 2023-10-16

**Authors:** Liangpin Li, Liyun Yuan, Kun Yang, Yanan Wu, Xia Hua, Yan Wang, Xiaoyong Yuan

**Affiliations:** 1 Clinical College of Ophthalmology, Tianjin Medical University, Tianjin, 300020 China; 2 Tianjin Key Laboratory of Ophthalmology and Visual Science, Tianjin Eye Institute, Tianjin Eye Hospital, Tianjin, 300020 China; 3 School of Medicine, Nankai University, Tianjin, 300071 China; 4 Tianjin Aier Eye Hospital, Tianjin University, Tianjin, 300190 China

**Keywords:** IOL power calculation formula, SMILE, FS-LASIK, Refractive error, Keratometry

## Abstract

**Background:**

As the two most prevalent refractive surgeries in China, there is a substantial number of patients who have undergone Femtosecond Laser-assisted In Situ Keratomileusis (FS-LASIK) and Small Incision Lenticule Extraction (SMILE) procedures. However, there is still limited knowledge regarding the selection of intraocular lens (IOL) power calculation formulas for these patients with a history of FS-LASIK or SMILE.

**Methods:**

A total of 100 eyes from 50 postoperative refractive surgery patients were included in this prospective cohort study, with 25 individuals (50 eyes) having undergone FS-LASIK and 25 individuals (50 eyes) having undergone SMILE. We utilized a theoretical surgical model to simulate the IOL implantation process in postoperative FS-LASIK and SMILE patients. Subsequently, we performed comprehensive biological measurements both before and after the surgeries, encompassing demographic information, corneal biometric parameters, and axial length. Various formulas, including the Barrett Universal II (BUII) formula, as a baseline, were employed to calculate IOL power for the patients.

**Results:**

The Barrett True K (BTK) formula, demonstrated an mean absolute error (AE) within 0.5 D for both FS-LASIK and SMILE groups (0.28 ± 0.25 D and 0.36 ± 0.24 D, respectively). Notably, the FS-LASIK group showed 82% of results differing by less than 0.25 D compared to preoperative BUII results. The Barrett True K No History (BTKNH) formula, which also incorporates measured posterior corneal curvature, performed similarly to BTK in both groups. Additionally, the Masket formula, relying on refractive changes based on empirical experience, displayed promising potential for IOL calculations in SMILE patients compared with BTK (*p* = 0.411).

**Conclusion:**

The study reveals the accuracy and stability of the BTK and BTKNH formulas for IOL power calculations in myopic FS-LASIK/SMILE patients. Moreover, the Masket formula shows encouraging results in SMILE patients. These findings contribute to enhancing the predictability and success of IOL power calculations in patients with a history of refractive surgery, providing valuable insights for clinical practice. Further research and larger sample sizes are warranted to validate and optimize the identified formulas for better patient outcomes.

## Introduction

Myopia is a significant global health concern, particularly prevalent in China and East Asia, where the estimated myopia rate among university students surpasses 90% [1–3]. Among this group, the main surgical methods for correcting myopia include corneal laser vision correction surgery and intraocular refractive surgery, with the former being more common in China [4, 5]. Two cutting-edge procedures in corneal laser vision correction (LVC) are Small Incision Lenticule Extraction (SMILE) and Femtosecond Laser-assisted In Situ Keratomileusis (FS-LASIK), which have gained widespread recognition for their remarkable outcomes in correcting myopia [6–10]. While the current refractive issues are resolved with these procedures, another problem that troubles ophthalmologists arises when these individuals develop cataracts. Due to significant changes in corneal shape, conventional IOL (Intraocular Lens) power calculation formulas become difficult to use accurately, often leading to substantial deviations. This seriously affects postoperative outcomes and restricts the use of multifocal IOL and other premium IOLs.

To address the issue of significant deviations in conventional formulas for post-refractive surgery patients, some researchers have improved these formulas in various ways, resulting in specialized formulas or options suitable for this group of patients [11, 12]. In light of the notable disparities observed in conventional formulas for patients who have undergone refractive surgery, several researchers have undertaken various approaches to address this issue. Consequently, specialized formulas or alternatives tailored to this specific patient cohort have been developed. Some of these formulas utilize regression algorithms, drawing from both past clinical data and empirical evidence to correct calculation outcomes. Conversely, other formulas integrate historical refractive data of patients both prior to and post-surgery into their algorithms [12]. Notably, the relatively limited duration of SMILE procedures and the younger age of the patients have resulted in a scarcity of substantial real-world clinical data. As a result, the development of the majority of these formulas has been primarily reliant on clinical data derived from LASIK and Photorefractive Keratectomy (PRK) procedures.

To assess the efficacy of different formulas after myopic refractive surgery and investigate their applicability to SMILE patients, we chose six formulas provided by ASCRS (three utilizing historical data and three without incorporating historical data). Subsequently, they conducted their investigations employing the virtual IOL implantation model proposed by Lazaridis and colleagues [13]. Moreover, we aimed to make a comparative analysis between the two techniques, offering valuable insights for managing a potentially substantial number of postoperative refractive patients in the future.

## Methods

This research project was approved by the Ethics Committee of Tianjin Eye Hospital and was conducted according to the principles of the Helsinki Declaration. This study enrolled patients with myopia who underwent FS-LASIK or SMILE surgery for refractive correction at Tianjin Eye Hospital between October 2022 and June 2023. Each patient was informed of the research content and signed the informed consent form before the surgery.

The patients were categorized into two groups: the FS-LASIK group and the SMILE group, depending on the specific surgical procedure they ultimately underwent. And all patients underwent ophthalmological evaluations preoperatively, which included assessments of uncorrected distance visual acuity, corrected distance visual acuity, manifest refraction, cycloplegic refraction, slit lamp examination, optical biometrics using IOL Master700 (Carl Zeiss Meditec AG, Jena, Germany), and corneal tomography with Pentacam HR (OCULUS Optikgeräte GmbH). Postoperative evaluations were conducted at least 3 months after the surgery and involved measurements of uncorrected distance visual acuity, corrected distance visual acuity, manifest refraction, and IOL Master700 assessments.

The inclusion criteria encompassed patients over 18 years of age with stable refraction for at least 2 years before the operation, corrected distance visual acuity of 20/20 or better, discontinuation of soft contact lens use for more than 2 weeks, and cessation of hard contact lens use for more than 4 weeks. Exclusion criteria consisted of any corneal or lens opacity or pathological changes observed during slit lamp examination, previous corneal surgery, ocular trauma or intraocular surgery, severe dry eye, glaucoma, corneal disease or ocular infection, keratoconus, or suspected keratoconus, remaining stromal thickness expected to be less than 280 µm, and posterior scleral staphyloma, among others.

The surgical procedures were conducted by a single experienced surgeon (Wang Y) utilizing the VisuMax femtosecond laser (Carl Zeiss Meditec AG, Jena, Germany). Throughout the surgeries, there were no recorded instances of intraoperative or postoperative complications.

In this study, the changes in intraocular lens (IOL) power before and after SMILE and FS-LASIK procedures were theoretically expected to be equal to the changes in corneal refractive power caused by the operations, as the lens and posterior segment of the eye are nearly unchanged. To simulate lens removal, a virtual surgical approach was adopted, and preoperative and postoperative optical biometry measurements were conducted using the IOL Master 700. For preoperative IOL power calculation, the Barrett Universal II (BUII) formula was utilized with the target refraction set as the refractive change diopter of the patient before SMILE. For example, if the preoperative spherical equivalent (SE) is -7.5 D, and the postoperative SE is -0.5 D, then the calculation would be as follows: -7.5 D - (-0.5 D) = -7 D. Postoperatively, the IOL power corresponding to a target refractive power of 0 D was calculated using six commonly used formulas, including Barrett True K (BTK), Masket, modified-Masket (M-Masket), Barrett True K no history (BTKNH), Shammas-PL, and Haigis-L formulas (The first three formulas utilize patient history data as parameters, while the latter three do not.), all of which were available at ASCRS online (https://ascrs.org/en/tools/post-refractive-iol-calculator). The calculation results were rounded to two decimal places, and the prediction error (PE) for each formula was determined by subtracting the preoperative BUII calculation result from each formula's calculation result. The absolute prediction error (AE) was obtained by taking the absolute value of PE. To ensure consistency in IOL power calculations, the same model of IOL (ZCB00, Johnson & Johnson Vision Care, Inc., A constant = 119.39) was chosen.

Descriptive statistics, including mean, standard deviation, and range, were computed using Microsoft Excel 2016. For the statistical analyses, we utilized SPSS software (Version 26.0, SPSS Inc., Chicago, IL, USA). Prior to analysis, data were assessed for normality using the Kolmogorov–Smirnov test, and the results were presented as mean ± standard deviation. The differences in axial length (AL), central corneal thickness (CCT), and white-to-white (WTW) measurements between the two groups were assessed using one-sample t-tests. The differences in anterior chamber depth (ACD) and mean keratometry (Km) were evaluated using the Mann–Whitney U test. To compare the differences in AE among the six formulas, one-way analysis of variance (ANOVA) was applied. For comparisons in sex distribution, the percentage of eyes falling within 0.5 D AE among the formulas, and the percentage of eyes falling within ± 0.25 D PE, Pearson's Chi-square test was used. A *p*-value less than 0.05 was considered statistically significant.

## Results

### Demographic and biometric data comparison

This study included 100 eyes from 50 postoperative refractive surgery patients, with 25 individuals (50 eyes) having undergone FS-LASIK (10 males, 15 females) and 25 individuals (50 eyes) having undergone SMILE (12 males, 13 females). Table 1 presents the demographic data and various corneal biometric parameters of the patients. The sex distribution was compared using Pearson's Chi-square test, revealing no statistically significant difference between the two groups (χ^2^ = 0.325, *p* = 0.569).

**Table 1:** Patient demographic and ocular biometry data before and after refractive surgery

	FS-LASIK group	SMILE group
Eyes/patients	50/25	50/25
Sex	M/F, 10/15	M/F, 12/13
Age (years)	25.04 ± 4.58 (18, 34)	26.00 ± 6.29 (18, 36)
AL (mm)		
Preop	25.93 ± 0.89 (23.76, 27.49)	25.6 ± 0.82 (23.79, 27.53)
Postop	25.79 ± 0.86 (23.71, 27.34)	25.5 ± 0.82 (23.86, 27.44)
ACD (mm)		
Preop	3.74 ± 0.30 (3.01, 3.37)	3.82 ± 0.32 (3.29, 4.48)
Postop	3.597 ± 0.26 (2.90, 4.06)	3.70 ± 0.29 (3.24, 4.27)
Km (D)		
Preop	43.86 ± 1.00 (41.89, 45.53)	43.34 ± 1.21 (36.22, 42.13)
Postop	38.54 ± 2.28 (40.88, 45.36)	39.65 ± 1.46 (36.94, 43.53)
SE (D)		
Preop	-5.78 ± 1.413 (-2.375, -11.125)	-4.63 ± 1.24 (-1.75, -8.25)
Postop	-0.46 ± 0.41 (0.5, -1.375)	-0.54 ± 0.32 (0.125, -1.5)
CCT (μm)		
Preop	535.4 ± 27.36 (485, 602)	546.9 ± 32.37 (494, 620)
Postop	428.2 ± 30.84 (377, 496)	475.0 ± 40.8 (413, 586)
WTW (mm)	12.22 ± 0.25 (11.60, 13.60)	12.19 ± 0.29 (11.50, 13.10)

Preoperative age and parameters such as AL, CCT, and WTW were compared between the two groups using t-tests after verifying normal distribution (results: t = 0.6169, *p* = 0.5402; t = 1.934, *p* = 0.056; t = 1.919, *p* = 0.579, respectively). Mann–Whitney U test was employed to compare ACD and Km between the groups (results: z = -1.093, *p* = 0.274; z = -1.948, *p* = 0.051, respectively). And no significant differences were found, hence, the two groups were well-matched in terms of age, sex, and corneal parameters.

### FS-LASIK group formulas comparison

**Fig. 1:**
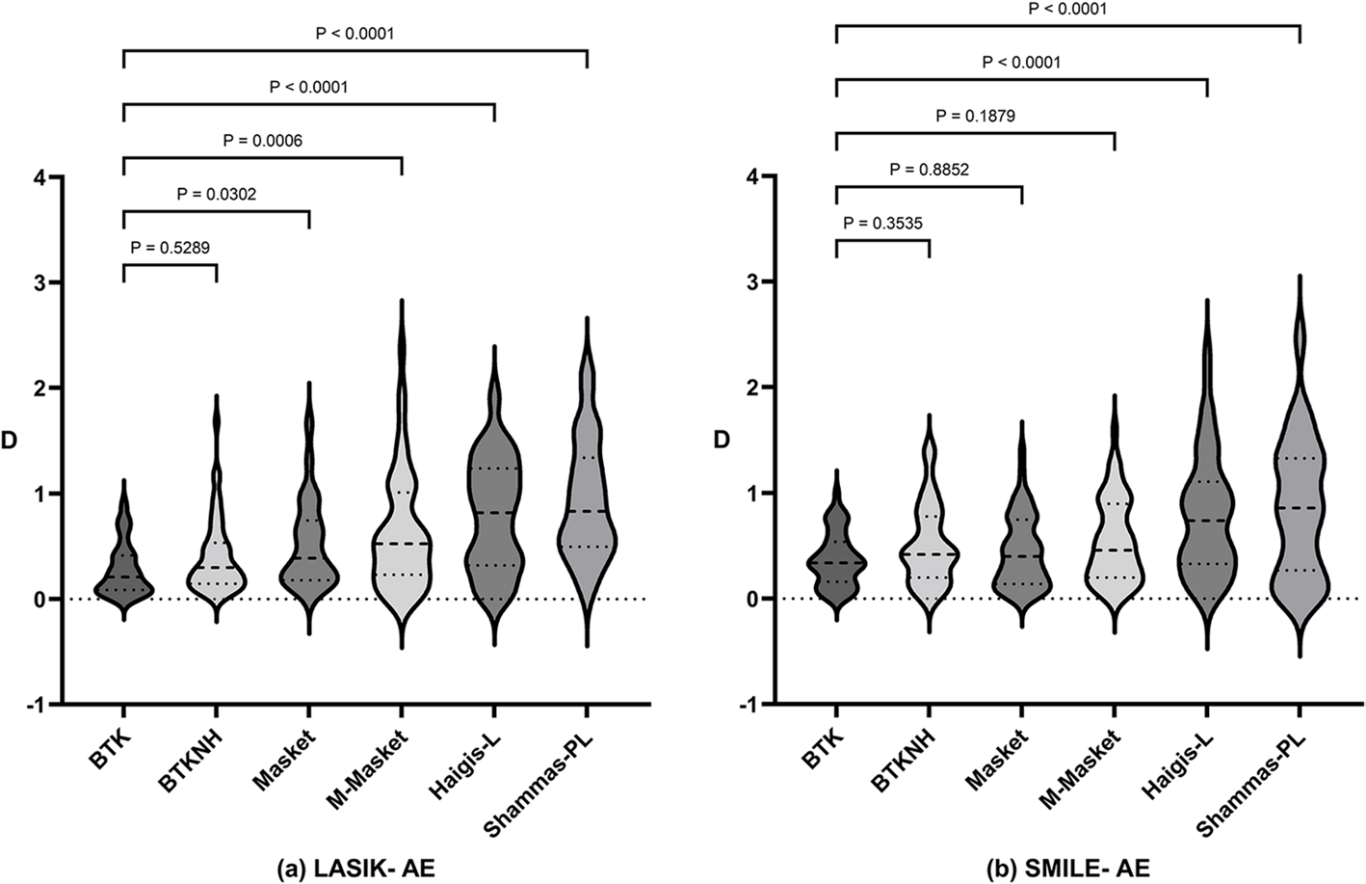
**a** Violin plot depicting the absolute prediction errors (AE) of 6 formulas in the LASIK group. **b** Violin plot depicting the AE of 6 formulas in the LASIK group. The formulas with the smallest mean AE were BTK (0.28 ± 0.25 D and 0.36 ± 0.24 D). In the LASIK group, BTK (0.89 ± 0.42 D) was significantly lower than the four other formulas except for BTKNH (0.40 ± 0.35 D). On the other hand, in the SMILE group, the mean AE of BTK was significantly lower compared to the Haigis-L and Shammas-PL formulas. However, there were no significant differences between BTK and the BTKNH (0.52 ± 0.37 D) and Masket (0.43 ± 0.33 D) formulas

Table 2 presents a comparison of the mean, standard deviation, extreme values, and median of the AE within the FS-LASIK group as generated by the six formulas. In the FS-LASIK group, the mean AE values for six formulas were as follows (in descending order): BTK (0.28 ± 0.25 D), BTKNH (0.40 ± 0.35 D), Masket (0.52 ± 0.41 D), M-Masket (0.63 ± 0.55 D), Haigis-L (0.80 ± 0.52 D), and Shammas-PL (0.94 ± 0.56 D). Figure 1a presents a Violin plot depicting the AE of six formulas in the FS-LASIK groups, and one-way ANOVA analysis indicated no significant difference between BTK and BTKNH (*p* = 0.18), but there were significant differences when compared to Masket, M-Masket, Shammas-PL, and Haigis-L formulas (*p* = 0.007, *p* < 0.001, *p* < 0.001, and *p* < 0.001, respectively).

**Table 2:** Absolute refractive prediction errors from BUII preoperative of FS-LASIK eyes

Formula	Mean	SD	min	max	median
BTK	0.27	0.25	0.00	0.92	0.20
BTKNH	0.39	0.35	0.00	1.68	0.30
Masket	0.51	0.41	0.00	1.70	0.74
M-Masket	0.62	0.55	0.00	2.37	0.52
Haigis-L	0.81	0.52	0.01	1.95	0.83
Shammas-PL	0.92	0.57	0.00	2.19	0.83

**Fig. 2:**
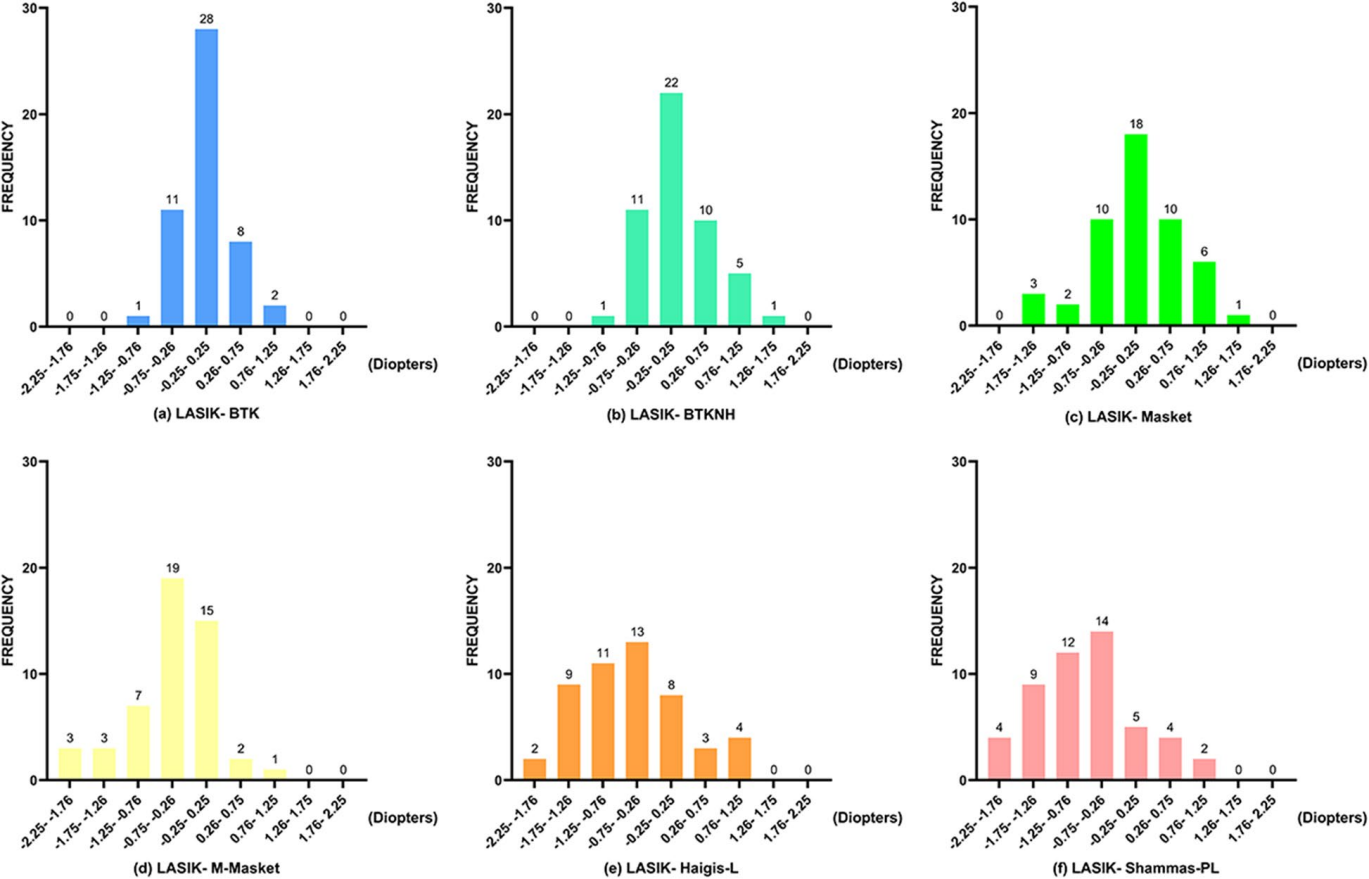
Frequency histogram of the prediction error of 6 formulas in the LASIK group. **a** The BTK formula; **b** the BTKNH formula; **c** the Masket formula; **d** the M-Masket formula; **e** the Haigis-L formula; **f** the Shammas-PL formula

Figures 2a-f and 4a and Table 3 illustrate the distribution frequency of AE and PE within various ranges for the six formulas. BTK (41 cases, 82%) and BTKNH (37 cases, 74%) had the highest proportion of AE within 0.5D, with no significant difference between them (χ^2^ = 0.932, *p* = 0.334). Remarkably, all AE results for BTK were within 1.0 D, indicating excellent stability. Additionally, BTK (28 cases, 56%) and BTKNH (22 cases, 44%) had the highest proportion of PE within ± 0.25 D, closely resembling BUII results, with no significant difference between the two (χ^2^ = 1.44, *p* = 0.23).

**Table 3:** The percentage of eyes falling within different ranges of Absolute refractive prediction errors among the formulas in LASIK group

Formula	± 0–0.5 D(%)	± 0.5–1.0 D(%)	± 1.0–1.5 D(%)	± 1.5–2.0 D(%)	> ± 2.0 D(%)
BTK	82.00	18.00	0.00	0.00	0.00
Masket	56.00	34.00	6.00	4.00	0.00
BTKNH	74.00	20.00	6.00	0.00	0.00
M-Masket	46.00	30.00	22.00	2.00	0.00
Haigis-L	38.00	22.00	32.00	8.00	0.00
Shammas-PL	26.00	36.00	18.00	14.00	6.00

### SMILE group formulas comparison

Table 4 presents a comparison of the key statistical measures for the AE within the SMILE group, as calculated using the six formulas. In the SMILE group, the mean AE values for the six formulas were as follows: BTK (0.36 ± 0.24 D), Masket (0.43 ± 0.33 D), BTKNH (0.52 ± 0.37 D), M-Masket (0.53 ± 0.39 D), Haigis-L (0.80 ± 0.56 D), and Shammas-PL (0.86 ± 0.65 D). Figure 1b illustrates the achieved AE of six formulas in the SMILE groups. The one-way ANOVA analysis demonstrated no significant difference between the BTK and Masket formulas, as well as between the BTK and BTKNH formulas (BTK-Masket: *p* = 0.411; BTK-BTKNH: *p* = 0.064). However, significant differences were found when comparing BTK with M-Masket, Shammas-PL, and Haigis-L formulas (*p* = 0.048, *p* < 0.001, and *p* < 0.001, respectively).

**Table 4:** Absolute refractive prediction errors from BUII preoperative of SMILE eyes

Formula	Mean	SD	min	max	median
BTK	0.37	0.25	0.01	1.00	0.34
Masket	0.44	0.34	0.00	1.39	0.40
BTKNH	0.51	0.37	0.00	1.42	0.42
M-Masket	0.54	0.39	0.01	1.59	0.46
Haigis-L	0.79	0.56	0.00	2.35	0.74
Shammas-PL	0.86	0.65	0.00	2.50	0.86

**Fig. 3:**
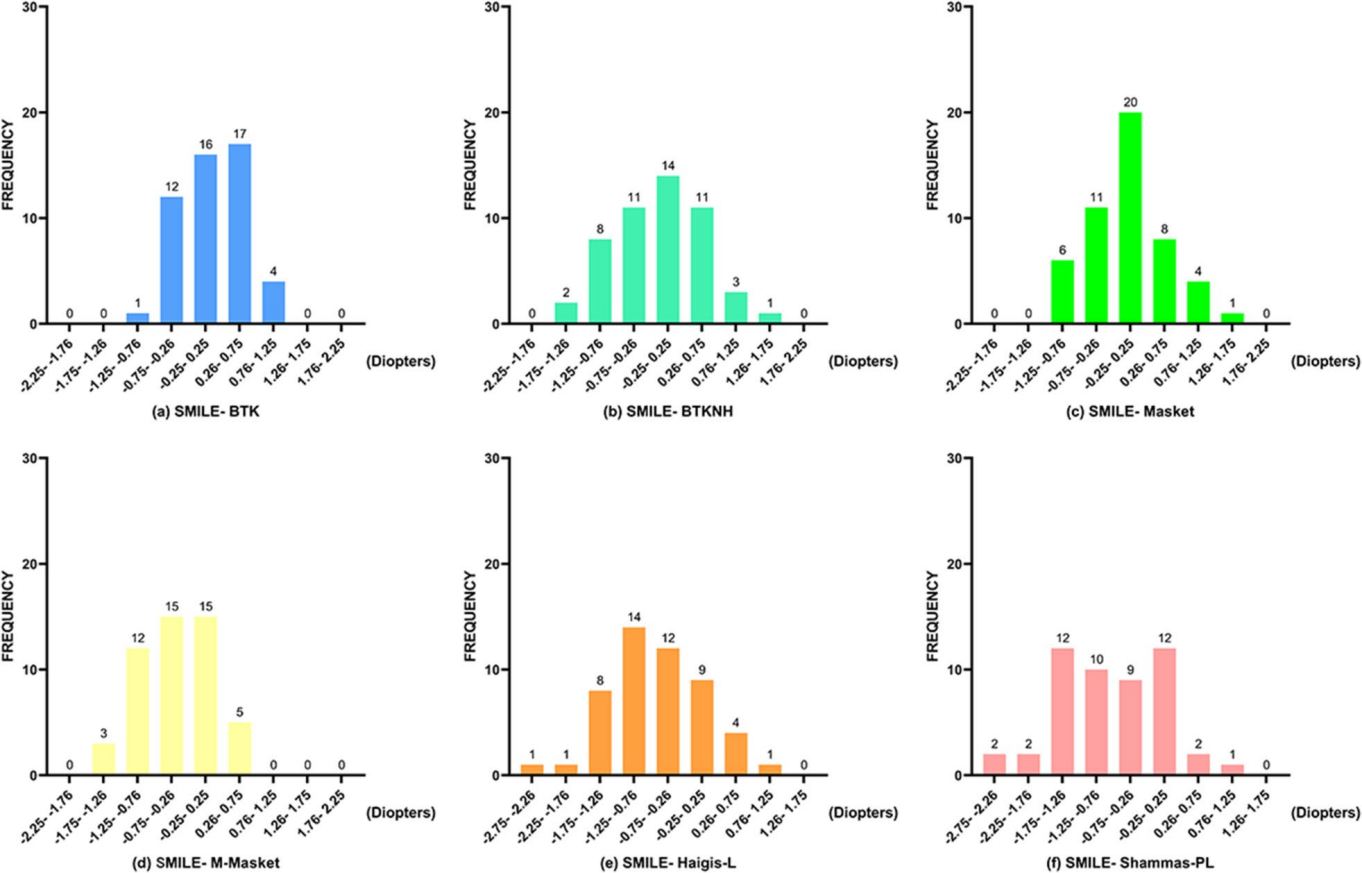
Frequency histogram of the prediction error of 6 formulas in the SMILE group. **a** The BTK formula; **b** the BTKNH formula; **c** the Masket formula; **d** the M-Masket formula; **e** the Haigis-L formula; **f** the Shammas-PL formula

**Fig. 4:**
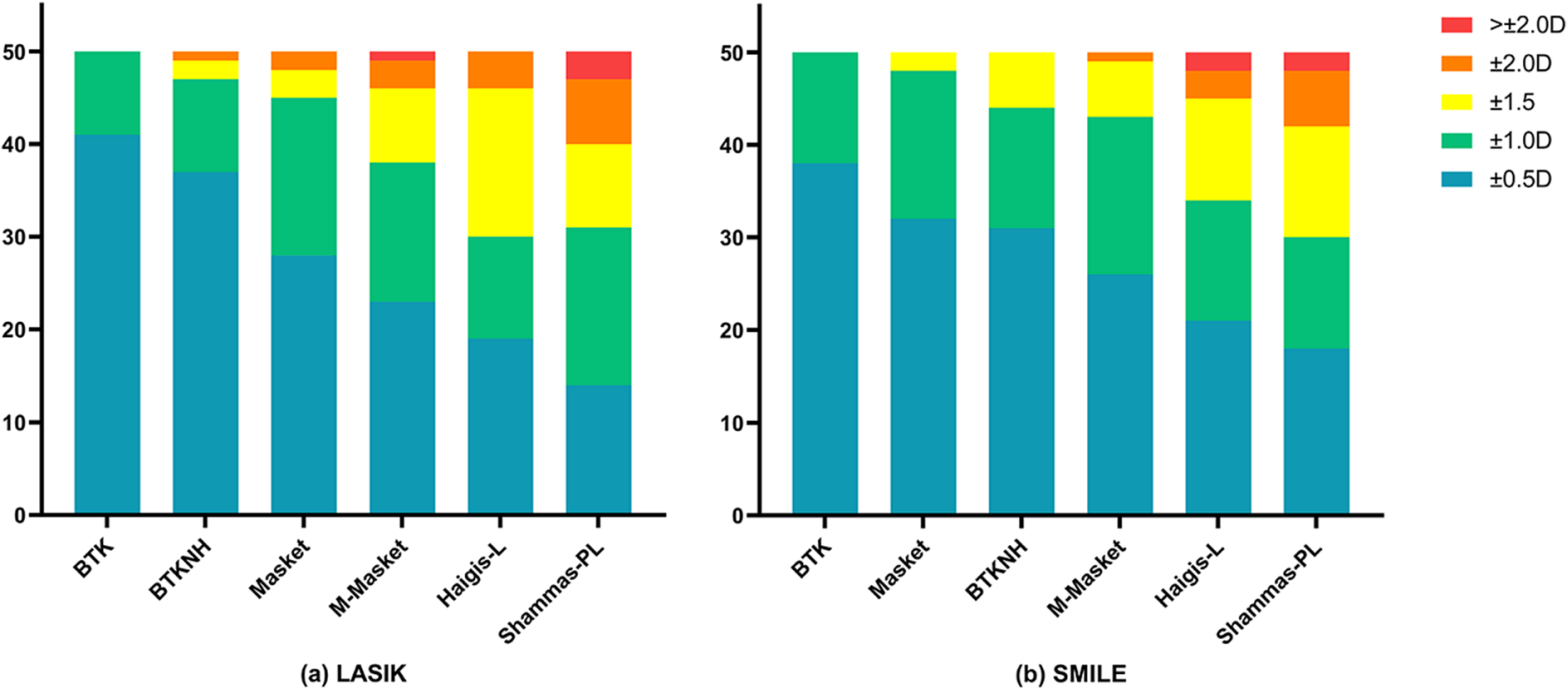
**a** The stacked histogram analysis compares the percentage of eyes within specific prediction error ranges with the preoperative BUII in the LASIK group. **b** The stacked histogram analysis compares the percentage of eyes within specific prediction error ranges with the preoperative BUII in the SMILE group. The BTK formula demonstrated favorable performance in both groups, with all results falling within ± 1.0 D. Additionally, a significant proportion of the outcomes, 82% for LASIK and 76% for SMILE, were within the range of ± 0.5 D

Similarly, Figs. 3a-f and 4b and Table 5 illustrate the distribution frequency of AE and PE within various ranges for the six formulas in the SMILE group. BTK (38 cases, 76%) had the highest proportion of AE within 0.5 D, followed by Masket (32 cases, 64%) and BTKNH (31 cases, 62%), with no significant difference between them (BTK-Masket: χ^2^ = 1.714, *p* = 0.19; BTK-BTKNH: χ^2^ = 2.291, *p* = 0.13). As in the FS-LASIK group, the BTK formula demonstrated excellent performance, with 100% of AE results within 1.0 D in the SMILE group. Moreover, the Masket formula had 96% of PE results within 1.0 D. Among the SMILE group, the formula with the highest proportion of PE within ± 0.25D was Masket (20 cases, 40%), and the chi-square test indicated a significant difference when compared to Haigis-L (Masket-Haigis-L: χ^2^ = 5.877, *p* = 0.015), but not when compared to other formulas (Masket-BTK: χ^2^ = 0.694, *p* = 0.405; Masket-BTKNH: χ^2^ = 1.604, *p* = 0.205; Masket-M-Masket: χ^2^ = 1.604, *p* = 0.205; Masket-Shammas-PL: χ^2^ = 2.941, *p* = 0.086).

**Table 5:** The percentage of eyes with absolute refractive prediction errors falling within various ranges was analyzed for the formulas used in the SMILE group

Formula	± 0–0.5 D(%)	± 0.5–1.0 D(%)	± 1.0–1.5 D(%)	± 1.5–2.0 D(%)	> ± 2.0 D(%)
BTK	76.00	24.00	0.00	0.00	0.00
Masket	64.00	32.00	4.00	0.00	0.00
BTKNH	62.00	26.00	12.00	0.00	0.00
M-Masket	52.00	34.00	12.00	2.00	0.00
Haigis-L	42.00	26.00	22.00	6.00	4.00
Shammas-PL	36.00	24.00	24.00	12.00	4.00

## Discussion

The improved surgical consistency, reduced cutting eccentricity, and minimized impact from surgeon-related factors [14, 15] allow for more accurate IOL power calculations. However, due to the relatively small number of cataract patients after refractive surgery, especially those undergoing SMILE, the widely accepted research approach currently involves using theoretical surgical models. This approach involves performing optical biometry on patients before and after refractive surgery to calculate the corresponding IOL power and to assess the accuracy of various formulas by calculating the corneal refractive changes caused by the surgical ablation [13, 16, 17].As a baseline, we used the preoperative results of the BUII formula [18], which is known for its exceptional accuracy and stability in the target population, especially for patients with medium to long axial lengths [19–21]. Our goal was to determine if the calculation accuracy in post-SMILE patients could match the performance of BUII in individuals without a history of refractive surgery. Such an outcome would be considered a significant success. Furthermore, another significant advantage of this model is that the follow-up intervals for patients are relatively short, and we intentionally retained various measurement information of the patients. As a result, when performing calculations, almost all patients' historical data is available for use.

With the support of historical data, the BTK formula demonstrated an average absolute error (AE) within 0.5 D for both groups, with FS-LASIK showing an impressive 82% of results differing by less than 0.25 D compared to the preoperative BUII results. The BTK formula which based on new-generation biometric devices capable of measuring the posterior corneal curvature, allowing for more accurate IOL power calculations that consider the influence of the posterior corneal curvature. Although the exact algorithm of the BTK formula has not been disclosed, it was observed in clinical practice that the formula primarily relies on preoperative manifest refraction and postoperative manifest refraction, without involving parameters like preoperative corneal K-values. On the other hand, BTK (including BTKNH) has an advantage in that it incorporates the measured posterior corneal curvature from machines like IOL Master 700, enabling more precise calculations of the total corneal curvature [22]. Studies, including this one, have shown that combining the posterior corneal curvature can enhance the accuracy of the BTK formula [23]. Another retrospective study by Savini and colleagues, involving 50 FS-LASIK surgery patients, also reached similar conclusions, indicating that using historical data and the posterior corneal curvature can improve the accuracy of the formula [24].

Presently, multiple studies have found that BTK performs well in FS-LASIK patients, but there is a lack of real case reports for SMILE patients. Zhu and co-workers' study simulated the surgery and found that BTK demonstrated high stability and consistency in both preoperative and postoperative SMILE patients, but it did not specify whether TK or SIMK values were used, nor whether the posterior corneal curvature was considered [17]. In this study, we found that both BTK and BTKNH performed similarly in both FS-LASIK and SMILE groups. However, in clinical practice, patients who can provide complete historical data are rare. Due to the lack of clinical consensus, the BTKNH formula, which relies more on empirical experience, has been more commonly used in previous studies. Ferguson and colleagues recently conducted a study involving postmyopic and post hyperopic eyes (FS-LASIK or PRK) and found that the BTKNH formula performed equivalently to a multiple formula approach on the ASCRS online calculator in both types of eyes [18]. Similarly, Abulafia and colleagues reached a consistent conclusion in their study involving 88 eyes (FS-LASIK or PRK) [25]. Another study by Lawless, which included 50 patients (72 eyes, FS-LASIK or PRK), not only found that the BTK formula performed better than other formulas but also discussed whether Total K or Sim K should be chosen in the formula [26]. As the IOL Master 700 currently calculates TK values from Sim K and PK values, most studies still input Sim K and PK values.

Interestingly, the Masket formula, which did not perform well in the FS-LASIK group, achieved results similar to BTK and BTKNH in the SMILE group, with the highest frequency of AE within 0.25D. Unlike BTK, the Masket formula partially discloses its algorithm, primarily relying on refractive changes based on empirical experience [27]. In previous research, several studies confirmed the reliability and accuracy of the Masket formula. Savini and colleagues' study, involving 22 patients (22 eyes, 15 of which had historical data), found that the Masket formula and Savini method produced the highest percentage of cases with an absolute prediction error in refraction of 0.50D in myopic-FS-LASIK patients with historical data [28]. Another study with a larger sample size (64 eyes, hyperopic-FS-LASIK) also found that the accuracy of the Masket formula was comparable to BTK when historical data was available [29]. However, the heavy reliance on historical data for result correction also limits the application of the Masket formula. Overall, there is still limited research on the Masket formula's use in IOL calculations after refractive surgery, and even fewer studies have been conducted on SMILE patients. However, the formula shows promising potential in SMILE patients. As for the M-Masket, Shammas-PL, and Haigis-L formulas, they did not perform well in both groups. The formula is a modified version of Masket with numerical adjustments, while Shammas-PL and Haigis-L are formulas that do not use historical data. BTKNH, which also does not use historical data, performed significantly better than Shammas-PL and Haigis-L in both groups. Additionally, these two formulas showed a trend of myopic drift, meaning their results tended to be higher than BUII. Despite this, they still produced consistent or similar results to BUII in some cases, making them potential reference options in clinical calculations.

Through this study, we find that patients with accessible historical data, wherein both FS-LASIK and SMILE procedures demonstrated a noteworthy proximity between the BTK formula and the preoperative BUII calculation results. This implies that the BTK formula may offer distinct advantages in IOL calculations for patients undergoing these two types of LVC surgeries. Moreover, among SMILE postoperative patients, the Masket formula exhibited commendable performance and serves as a crucial point of reference. Furthermore, in cases where historical data is unavailable, the BTKNH formula emerges as a more dependable alternative.

## Data Availability

The datasets generated during and/or analyzed during the current study are available from the corresponding author on reasonable request.
